# Spatial Transcriptomics in Breast Cancer: Advances and Applications

**DOI:** 10.3390/biology15131061

**Published:** 2026-07-03

**Authors:** Yanni Cao, Kangcheng Xu, Xiaohui Li, Junyuan Zhang, Wen Jin, Yuxian Liu

**Affiliations:** 1 School of Artificial Intelligence, Anhui University of Science & Technology, Huainan 232001, China; cyn@aust.edu.cn (Y.C.);; 2 State Key Laboratory of Digital Intelligent Technology for Unmanned Coal Mining, Anhui University of Science & Technology, Huainan 232001, China; 3 Clinical Medical Research Center, Inner Mongolia People’s Hospital, Hohhot 010010, China

**Keywords:** breast cancer, spatial transcriptomics, tumor microenvironment, tumor heterogeneity, precision medicine

## Abstract

Background/Objectives: While traditional transcriptomics and single-cell RNA sequencing can reveal differences in cell type and gene expression, they cannot provide spatial information within tissues. Spatial transcriptomics (ST), as an emerging technology in recent years, has achieved significant progress in resolving gene expression along the spatial dimension. This technology quantifies gene expression at defined spatial coordinates and describes the spatial distribution of transcripts and the co-localization patterns between cells within intact tissue, allowing for an integrated analysis of molecular and spatial information. This review aims to systematically trace the development of ST and highlight its application value in breast cancer research. Methods: We systematically reviewed the recent literature on ST platforms, on combined analyses of single-cell RNA sequencing (scRNA-seq) and ST, and on integrated spatial multi-omics in breast cancer. Key topics include tumor microenvironment organization, intra-tumor heterogeneity, the spatial distribution of immune cells, cancer-associated fibroblast function, treatment-response prediction, and personalized-treatment strategy development. Results: ST can characterize the spatial organization of interactions between breast cancer cells and the tumor microenvironment, describe the spatial dimensions of tumor heterogeneity, and provide multi-dimensional information that may support refined subtype classification and prognostic assessment. Existing studies indicate that ST shows significant potential to inform personalized treatment strategies, but the technology also faces bottlenecks in data integration, spatial resolution, standardization, and the need for functional validation. Conclusions: ST provides an important tool for an in-depth description of the complex spatial organization within breast cancer tumors. When integrated with functional perturbation, longitudinal cohorts, and orthogonal omics, it has the potential to ultimately improve clinical outcomes for breast cancer patients.

## Simple Summary

While traditional gene sequencing technologies can identify cell types and gene activity, they cannot tell us where these cells live or how they are organized within tumor tissue. Spatial transcriptomics, an emerging technology, can simultaneously record gene expression information at each location while preserving the spatial structure of the tissue, essentially creating a detailed “gene map” for the tumor. This review systematically traces the development of this technology and focuses on its application in breast cancer research. Studies have shown that spatial transcriptomics can characterize the spatial organization of interactions between breast cancer cells and their surrounding “supporting environment,” describe differences between regions within the same tumor, and help identify the distribution patterns of immune cells and supporting cells. This information may help to refine breast cancer classification and prognostic assessment, and provide a scientific basis for developing personalized treatment strategies. Although this technology still faces certain challenges in data processing, spatial resolution, and the need for functional validation, it shows great potential to support precision medicine for breast cancer and, ultimately, to contribute to improving patients’ quality of life.

## 1. Introduction

According to GLOBOCAN estimates released by the International Agency for Research on Cancer, on average, one in every 20 women worldwide will develop breast cancer, and projections indicate that by 2050 there will be approximately 3.2 million new cases of breast cancer and 1.1 million breast cancer-related deaths annually [1,2]. Breast cancer has therefore become a major public health issue worldwide. Current clinical management is guided by molecular subtyping and combines surgery, radiotherapy, chemotherapy, endocrine therapy, HER2-targeted therapy, and, more recently, immunotherapy, in line with successive NCCN guideline updates [3,4]. Despite these advances, the five-year survival rate for patients with advanced disease remains below 30%, and triple-negative breast cancer (TNBC)—which represents roughly 15% of all breast cancers—continues to show high rates of early recurrence, with cumulative recurrence reported in 30–40% of patients within the first five years of follow-up [5]. These figures highlight a clinical landscape in which improvements in early detection and systemic therapy coexist with a substantial unmet need in advanced and triple-negative disease.

Closing this clinical gap requires a more granular molecular description of breast cancer tissue than current routine approaches can provide. Bulk RNA sequencing yields population-averaged transcriptomes that obscure intra-tumor heterogeneity and discard the in situ position of every cell [6,7]. Single-cell RNA sequencing (scRNA-seq) restores cellular resolution but requires tissue dissociation, which destroys the native tissue architecture and the spatial coordinates of cell–cell contacts [8]. Immunohistochemistry (IHC)-based subtyping preserves morphology and reports a small number of canonical markers (ER, PR, HER2, Ki-67), but it samples only a handful of proteins and cannot resolve the global transcriptional state of the microenvironment surrounding each tumor region [9,10]. The combined effect of these three limitations—loss of spatial information in bulk assays, loss of in situ context in scRNA-seq, and limited molecular breadth in IHC—leaves a structural blind spot precisely where breast cancer biology is most heterogeneous: in the spatially organized interactions between malignant cells, immune infiltrate, stromal fibroblasts, vasculature, and extracellular matrix within primary and metastatic lesions.

**Figure 1:**
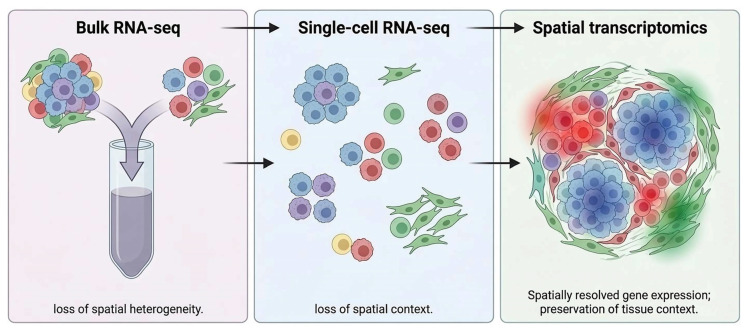
Advantages of spatial transcriptome technology compared with other technologies. Arrows show the comparison from bulk RNA-seq to single-cell RNA-seq and spatial transcriptomics. Different colors represent different cell populations.

Spatial transcriptomics, as an emerging technology that combines gene expression information with tissue spatial location information, allows for the systematic analysis of transcriptional characteristics in different spatial regions of tissue sections while preserving tissue morphology and structure [11]. By incorporating spatial context, ST provides a novel research framework for analyzing tumor heterogeneity, cell–cell interactions, and the functional compartmentalization of the tumor microenvironment, thereby significantly expanding the explanatory dimensions of traditional transcriptomics in cancer research [12,13]. Furthermore, spatial transcriptomics integrates transcriptomic information with spatial structure at the tissue level, providing unique advantages for cancer research compared to traditional bulk RNA sequencing and single-cell sequencing (Figure 1) [14]. Based on this, this paper systematically reviews the recent development of spatial transcriptomics technology and its applications in breast cancer research, focusing on summarizing the latest progress in understanding tumor heterogeneity and the tumor microenvironment. At the same time, this paper further explores the potential applications of spatial transcriptomics in the personalized precision treatment of breast cancer, evaluates its clinical translation prospects, and analyzes the challenges and limitations currently faced by this technology.

## 2. Principles and Development of Spatial Transcriptomics

As a groundbreaking technology in the field of genomics, spatial transcriptomics enables researchers to analyze the spatial distribution of gene expression whilst preserving the structural integrity of tissue [15]. The concept of this technology can be traced back to in situ hybridization research in the late 1970s, but its development was constrained by the technical limitations of the time [16]. With the maturation of high-throughput sequencing technology and the gradual exposure of the limitations of traditional gene expression analysis methods in terms of spatial resolution, such as the averaging of expression data between samples, leading to loss of spatial resolution and blurred biological insights [17]. Against this backdrop, spatial transcriptomics has experienced rapid development over the past decade. This technology, by combining transcriptome data with spatial coordinate information, can finely characterize the composition, heterogeneity, and interactions of cells in biological tissues, thus largely overcoming the limitations of single-cell RNA sequencing (scRNA-seq) [18,19].

Currently, spatial transcriptomics technology can be divided into four categories: imaging hybridization, capture sequencing, in situ sequencing, and spatial multi-omics.

### 2.1. Imaging-Based Spatial Transcriptomics (smFISH, MERFISH, seqFISH+)

Imaging-based spatial transcriptomics can be applied in in situ hybridization (ISH) chemistry to describe fluorescently labeled oligonucleotide probes that bind complementary mRNA sequences to localize transcripts directly within intact tissue sections [20]. In spatial transcriptomics, this method combines the spatial structure of tissue sections with gene expression information, allowing researchers to directly observe the spatial distribution patterns of genes in tissues through high-resolution microscopy imaging [21]. Single-molecule FISH (smFISH) extends conventional ISH by tiling tens of short oligonucleotide probes along each transcript, yielding diffraction-limited single-mRNA detection and bringing sensitivity close to one copy per cell [22]. The two dominant high-multiplex evolutions of smFISH are MERFISH and seqFISH+: both encode each gene with a binary or base-q barcode read-out across sequential rounds of hybridization and imaging, raising panel size from a handful of genes to ~1000–10,000 transcripts per experiment while retaining subcellular (≈100–250 nm) spatial resolution [23]. Commercial implementations—Vizgen MERSCOPE, NanoString CosMx SMI, and 10x Genomics Xenium—operate on the same imaging-cycle principle and are compatible with both fresh-frozen and FFPE sections, supporting tissue areas of ≈1 cm^2^ per run. In breast cancer, imaging-based ST has been used to (i) map HER2 heterogeneity and HER2-low subpopulations at single-cell resolution in invasive ductal carcinoma [24], (ii) resolve tumor–immune cell neighborhoods around tertiary lymphoid structures in triple-negative breast cancer [25], and (iii) co-localize epithelial-to-mesenchymal-transition (EMT) transcripts with stromal niches in lobular carcinoma [24]. The principal limitation of imaging-based ST is its targeted nature: the gene panel must be pre-designed, optical crowding caps achievable multiplexing below the full transcriptome, and long multi-round imaging schedules constrain throughput.

### 2.2. Capture Sequencing Technology

Capture-based ST anchors spatially barcoded oligo-dT or gene-specific capture probes to a defined substrate; permeabilized tissue is laid on the substrate so that released mRNAs diffuse onto the nearest probes, after which the captured molecules are converted to a sequencing library whose reads can be mapped back to their tissue-of-origin coordinate via the barcode [26,27]. Three platform families dominate current practice. 10x Genomics Visium uses 55 µm spots arrayed on a 6.5 × 6.5 mm capture area with a 100 µm center-to-center pitch, supports both fresh-frozen and FFPE sections, and provides whole-transcriptome coverage at multi-cell resolution [28]. Slide-seqV2 and the open-source Stereo-seq platform push the substrate-level resolution to ≈10 µm and below, at the cost of lower per-spot capture sensitivity and increased computational demand [29,30,31,32]. Microfluidic-deterministic-barcoding platforms such as DBiT-seq use orthogonal channel-grid barcoding to achieve ~10 µm pixel resolution and to enable the multi-modal joint capture of mRNA, protein, and chromatin tags from the same section [30]. In breast cancer, capture-based ST has been applied to (i) profile the spatial architecture of ductal carcinoma in situ (DCIS) and its progression to invasive disease [33], (ii) map cancer-associated fibroblast subtypes and their juxtaposition with malignant clones in HER2+ and TNBC tumors [34], and (iii) integrate spatial expression with bulk-cohort survival data to identify prognostic niches [35]. The principal limitations of capture-based ST are its sub-cellular spatial resolution (one Visium spot typically covers 1–10 cells, blurring rare-cell signals through deconvolution), lateral mRNA diffusion during permeabilization (which can broaden the apparent spatial footprint of a transcript by 10–50 µm and is the dominant source of edge-of-spot artifacts), and reduced sensitivity for low-abundance transcripts relative to imaging-based approaches.

### 2.3. In Situ Sequencing Technology

In situ sequencing (ISS) reads short transcript-derived tags directly inside the tissue section. Padlock probes are circularized on the target mRNA, rolling-circle-amplified into nanoball clusters, and sequenced through successive interrogations of base-specific fluorophores using microscopy rather than a flow cell [3]. Two complementary subfamilies are in active use. Targeted ISS—including the original Ke and colleagues’ chemistry, HybISS, and BaristaSeq—typically reads gene-identifier barcodes for 50–500 pre-selected transcripts at ~250 nm subcellular resolution on fresh-frozen or FFPE sections [22]. Untargeted ISS—including FISSEQ and STARmap/STARmap PLUS—sequences the transcript itself, providing access to short-read–level sequence variation at the cost of lower throughput and higher computational burden [36,37]. Section-level fields of view are typically ≈1 cm^2^ and 1–5 mm thick for STARmap PLUS, supporting whole-tissue analyses of small biopsies [38]. In breast cancer, ISS has been used to (i) map clonal architecture and copy-number heterogeneity in DCIS and invasive ductal carcinoma at single-cell resolution, (ii) localize HER2 and ESR1 transcripts together with immune-checkpoint mRNAs to define spatially resolved targetable niches [24]. The principal limitations of ISS are its dependence on long multi-round imaging schedules (typically 6–10 cycles for targeted ISS, 30+ for untargeted ISS), optical crowding at high transcript density, and a steep computational cost for image registration, base calling, and spot decoding across cycles.

### 2.4. Spatial Multi-Omics Technology

Spatial multi-omics technologies pair ST with a second molecular layer measured on the same—or a serially registered—tissue section, with the aim of moving from a transcript-only view of the tumor microenvironment toward a joint readout of transcription, translation, post-translational state, and metabolite distribution [39,40,41]. Three families have matured into demonstrated case studies in breast cancer.

**(i) ST + Spatial Proteomics:** Co-registered Visium + CODEX (PhenoCycler-Fusion) and Visium + imaging mass cytometry (IMC) workflows have been applied to invasive breast carcinoma to pair transcript-level state with 40–60-marker protein panels, allowing for direct testing of whether transcript abundance predicts the post-translational immune-checkpoint and signaling-pathway state in the same niche [42,43,44].

**(ii) ST + Spatial Metabolomics:** Visium or Stereo-seq sections paired with adjacent-section MALDI-MSI (matrix-assisted laser desorption/ionization mass spectrometry imaging) or DESI-MSI have been used to overlay transcripts of lipid-metabolism and one-carbon-metabolism pathways with the spatial distribution of their substrates and products in breast cancer, identifying transcript–metabolite mismatches that motivate functional follow-up [45,46].

**(iii) ST + Spatial Epigenomics:** Spatial-ATAC-seq, spatial-CUT&Tag, and the joint spatial RNA + ATAC platform MISAR-seq couple chromatin-accessibility or histone-mark mapping to ST on the same section; emerging applications in breast cancer focus on subtype plasticity, endocrine-resistance reprogramming, and the regulatory state of cancer-associated fibroblasts [47,48,49,50,51].

Four technical challenges currently limit the field. First, FFPE compatibility is uneven: spatial proteomics imaging is mature for FFPE, but most spatial-metabolomics and spatial-epigenomics chemistries still require fresh-frozen sections, constraining clinical-archive use. Second, the modalities differ by an order of magnitude in native spatial resolution (ST 2–55 µm, IMC/CODEX subcellular, MSI 5–50 µm), so cross-modality alignment requires either careful section-to-section registration or downsampling, each of which introduces information loss. Third, joint analyses must contend with modality-specific batch effects whose magnitude can exceed the biological effect of interest, particularly in multi-center cohorts. Fourth, there is no consensus benchmark for evaluating multi-omics integration in tissue: methods such as PASTE, GLUE, MOSCA, and SpaMosaic have been proposed but have not been compared on a community-curated breast-cancer ground-truth dataset [40].

The analytical bottleneck is therefore no longer data acquisition but principled integration: end-to-end inference of cell-type, state, regulatory, and metabolic layers from co-registered modalities, with explicit quantification of integration uncertainty, remains an open problem that limits the translational use of spatial multi-omics in breast cancer.

**Figure 2:**
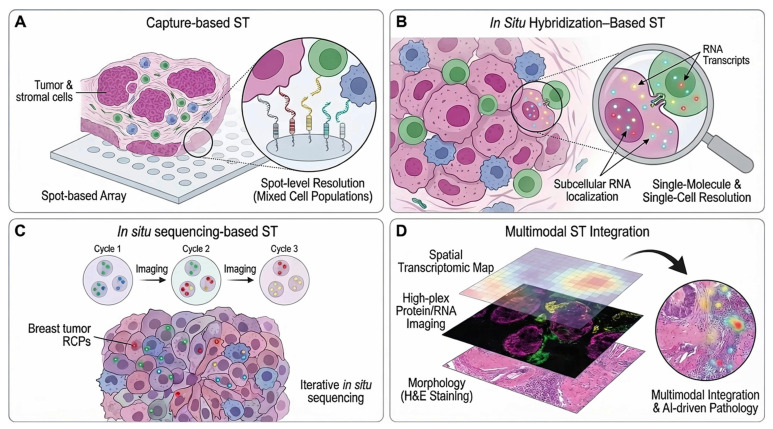
Main technical strategies of spatial transcriptomics. (**A**) Capture-based ST uses spot-based arrays and provides spot-level resolution. (**B**) In situ hybridization-based ST localizes RNA transcripts at subcellular or single-cell resolution. (**C**) In situ sequencing-based ST reads RNA signals through iterative imaging cycles. (**D**) Multimodal ST integrates spatial transcriptomic maps with protein/RNA imaging and tissue morphology. Arrows indicate the assay workflow in each panel. Different colors represent different cell populations, RNA species, or molecular modality layers.

Overall, existing spatial transcriptomics technologies can be grouped into capture-based, in situ hybridization-based, in situ sequencing-based, and emerging imaging-based or multimodal platforms, which differ in spatial resolution, transcriptome coverage, and the scope of molecular information they provide (Figure 2; see Table 1 for a side-by-side comparison).

Despite this rapid platform diversification, four cross-cutting technical challenges constrain every current ST family and shape the trade-offs summarized in Table 1. First, there is an unavoidable resolution-versus-coverage trade-off: capture-based platforms achieve near-whole-transcriptome coverage but at multi-cell spot sizes, while imaging-based platforms reach subcellular resolution only after committing to a pre-defined gene panel, and pushing capture-based resolution toward 1–2 µm (e.g., Visium HD, Stereo-seq) sharply reduces per-bin capture sensitivity. Second, lateral mRNA diffusion during permeabilization in capture-based workflows can broaden the apparent spatial footprint of a transcript by 10–50 µm, generating edge-of-spot artifacts that propagate into downstream cell-type deconvolution and spatial-domain calls. Third, optical crowding in highly multiplexed FISH-based platforms places a practical ceiling on panel size before adjacent single-molecule spots become indistinguishable; this ceiling, together with the multi-round imaging time, sets the upper bound on imaging-based throughput. Fourth, the field still lacks consensus quality-control standards: there is no agreed metric for spatial integration accuracy, no community-curated breast-cancer ground-truth dataset spanning platforms, and no standardized reporting checklist for pre-analytical and analytical parameters. We return to these challenges, together with their computational and clinical implications, in Section 5.

### 2.5. Spatial Transcriptomics in the Context of Other Spatial Modalities

Spatial transcriptomics is not the only modality that preserves the spatial coordinate of a molecular measurement. A parallel family of high-multiplex spatial proteomics imaging platforms has matured over the same period and now provides comparable subcellular resolution at the protein level. Imaging mass cytometry (IMC, the CyTOF-imaging derivative) and multiplexed ion-beam imaging (MIBI/MIBIscope) read 40–60 metal-isotope-tagged antibodies per section using mass-spectrometry-based detection, while CODEX/Akoya PhenoCycler-Fusion uses cyclic fluorescence imaging of oligonucleotide-barcoded antibodies to reach similar panel sizes on FFPE sections at scales compatible with clinical archives [57]. Recent breast-cancer studies illustrate the maturity of this complementary axis: a 2024–2025 multi-platform analysis combined CycIF, IMC and MIBI on 102 ER+ breast-cancer patients to identify prognostic immune and stromal spatial biomarkers [44], and a 2024 Br J Cancer review provides a cross-platform comparison of multiplexed imaging technologies for cancer-tissue characterization [58].

The choice between ST and spatial proteomics imaging is governed by the scientific question rather than by an absolute hierarchy. ST is preferred when (i) the goal is unbiased, transcriptome-wide discovery or hypothesis generation in a poorly characterized niche, (ii) the target list is not yet defined, or (iii) splice-isoform, mutation, or non-coding RNA information is required. Spatial proteomics imaging is preferred when (i) the post-translational functional state of a small, well-defined panel of proteins is the readout of interest (phosphorylation, cytokine production, immune-checkpoint surface expression), (ii) FFPE-archive compatibility and turnaround times consistent with clinical workflows are decisive, or (iii) subcellular resolution at a fixed cost is needed. Spatial multi-omics (see Section 2.4) is appropriate when both molecular layers are required to answer the question and the experimental and analytical overhead of joint acquisition is justified. A detailed comparison of ST with the principal spatial proteomics imaging modalities used in breast cancer is summarized in Table 2.

In practice the two families are increasingly used as complements rather than alternatives. Co-registered ST + CODEX and ST + IMC workflows on adjacent or serial sections have been applied in breast cancer to test, on the same niche, whether transcript-level signatures predicted by ST are recapitulated at the protein level and whether the inferred cell-type composition produced by ST deconvolution matches the directly imaged proteomic composition [57]. Such pairings provide cross-modality validation, supply an external ground truth for benchmarking deconvolution and spatial-domain detection methods, and represent the most realistic near-term route to clinically useful spatial assays.

## 3. Research Progress of Spatial Transcriptomics in Breast Cancer

With the rapid development of spatial transcriptomics (ST) technology, it has been increasingly applied to breast cancer research, providing a new research perspective for understanding the complex biological characteristics of breast cancer. Currently, the main clinical treatment methods for breast cancer is a comprehensive approach based on molecular subtyping, integrating surgery, radiotherapy, chemotherapy, endocrine therapy, targeted therapy, and immunotherapy. However, breast cancer patients, especially those at advanced stages, still face a high risk of recurrence and poor clinical prognosis. This situation largely stems from the significant heterogeneity of the tumor microenvironment (TME), which in turn induces treatment resistance and immune escape in tumors. The tumor microenvironment is composed of tumor cells, immune cells, fibroblasts, vascular endothelial cells, extracellular matrix, and various secretory factors. Traditional studies typically view the TME as a homogeneous whole, but increasing evidence suggests that spatial functional partitioning exists within the TME—different regions possess distinctly different cellular compositions, signaling networks, and metabolic characteristics, forming specific “spatial niches”. Such spatial heterogeneity is associated with the adaptive evolution of tumor subclones, the establishment of immunosuppressive microenvironments, and the development of treatment resistance. Therefore, elucidating the spatial organization structure and regulatory mechanisms of the tumor microenvironment is of great research value. Conventional bulk sequencing loses spatial location information, while single-cell sequencing destroys the in situ structure of tissues. Neither of these methods can systematically resolve the causal relationship between TME spatial heterogeneity and clinical drug resistance. In contrast, spatial transcriptomics technology can combine transcriptome data with spatial location information to obtain intercellular interactions while preserving the integrity of tissue structure. The following section will systematically summarize the latest advances in spatial transcriptomics technology in breast cancer research, focusing on its application value in tumor microenvironment analysis, tumor heterogeneity analysis, and research on treatment resistance mechanisms.

### 3.1. Spatial Transcriptomics in Tumor Microenvironment

The tumor microenvironment comprises tumor cells, immune cells, stromal cells, vascular networks, and extracellular matrix, and its spatial heterogeneity and dynamic interactions exert a substantial influence on tumor progression and treatment response [61,62,63]. Spatial transcriptomics, by combining high-resolution imaging with transcriptome-wide profiling, has revealed multi-level features of the breast-cancer TME that inform the formulation of precision treatment strategies [2,54,64,65]. Because the broader advantages of ST over bulk and single-cell RNA-seq are introduced in Section 2 and revisited only where they directly motivate a specific finding, the present section concentrates on what ST has actually added to TME biology. The recurrent spatial niches identified by ST in the breast-cancer tumor microenvironment are summarized in Table 3.

#### 3.1.1. Advantages of ST in TME

Unlike the averaged readout of bulk transcriptomics, spatial transcriptomics captures regional gene-expression heterogeneity within tumors. In breast cancer, ST has been used to identify spatially restricted tumor subpopulations and signaling-pathway differences, enabling near-single-cell mapping of how tumor cells interact with stroma, vasculature, and immune compartments [66]. Representative observations include the following. In the tumor-margin region, fibroblast-derived EMILIN1 is spatially co-localized with CD8+ T-cell infiltration, raising the possibility that EMILIN1+ fibroblasts contribute to T-cell recruitment via short-range proximity effects; its expression intensity correlates positively with favorable prognosis [64,67,68]. Such spatial correlations remain hypothesis-generating, and parallel functional work using orthotopic models and perturbation assays will be required to establish a causal contribution [64].

ST can also be used to build three-dimensional correlative models that link transcriptome and spatial-coordinate data, supporting descriptive analyses of multiple biological processes within the TME [4,69]. Reported applications include the following. For functional gene localization, ST has been used to pinpoint the expression territories of immune-related genes such as CTLA4 and PD-L1 and to describe their spatial association with immune-cell infiltration and matrix fibrosis [70]. For cell-interaction analysis, in HER2+ breast cancer ST has shown that the spatial distribution of tumor-associated fibroblasts co-localizes with immunosuppressive cytokines and has identified an iCAF-like subset whose high FAP expression is associated with an epithelial–mesenchymal-transition (EMT) signature at the invasion front, although the causal role of these fibroblasts in EMT will require dedicated perturbation studies [71]. For biomarker discovery, ST has nominated spatially enriched candidates in breast cancer, such as SREBF1 in invasive micropapillary carcinoma (IMPC) [72].

For immune-infiltration analysis, ST can quantify the spatial distribution and distance-dependent neighborhood relationships of immune-cell subsets (T cells, macrophages, and others) and is being used to characterize prognostically relevant immune phenotypes [73]. In triple-negative breast cancer (TNBC), the spatial proximity of intratumoral lymphocytes to cancer nests is significantly associated with survival, with the high-ITL group showing a markedly higher 5-year survival rate than the low-ITL group [74]. By integrating morphology with transcriptomic readouts, ST has further described high-expression territories of CTLA4 and PD-L1 in the tumor core that spatially co-occur with M2-polarized macrophage aggregates and with regions depleted of cytotoxic T cells, a pattern consistent with, although not direct proof of, local immune exclusion [75,76].

ST also provides a spatial readout of tumor-cell metabolism. For example, the heterogeneous expression of the glycolytic gene LDHA in the invasive region is consistent with the hypothesis that marginal-zone cancer cells engage an oxidative-phosphorylation (OXPHOS) metabolic switch during dissemination, and may motivate dedicated functional validation in patient-derived models [77]. This spatial-localization-to-candidate-mechanism workflow has nominated spatially enriched biomarkers, including SREBF1 in invasive micropapillary carcinoma (IMPC) and a margin-restricted CXCL12-CXCR4 signaling axis in triple-negative breast cancer [78]. Follow-up perturbation studies, such as the autophagy-coupled lipid-metabolism work of Geng and Guo (2024) [71], illustrate the type of in vitro and in vivo validation that is now needed to convert these spatial associations into actionable mechanisms.

The integration of ST technology with single-cell sequencing and spatial proteomics has enabled TME analysis to move from a single dimension to multi-omics analysis. For example, by combining laser microdissection with ST-seq technology, a transitional cell population with high CXCR3 expression was identified at the tumor–immune junction of breast cancer. These cells specifically mediate the spatial recruitment of immune cells [79,80,81].

In summary, spatial transcriptomics can describe the spatial layout and putative interactions of cells in the tumor microenvironment, providing spatial context that supports the understanding of tumor biology and the design of targeted therapies. This illustrates the contribution of spatial transcriptomics to unraveling the complex tumor microenvironment.

#### 3.1.2. Progress of ST in Immune Microenvironment

Spatial transcriptomics provides an unprecedented spatial perspective for analyzing the tumor immune microenvironment (TIME). By precisely locating the spatial distribution and functional state of immune cells, ST reveals the distribution patterns of tumor-infiltrating lymphocytes (TILs) in tumor tissues [67,82]. In breast cancer research, ST found that the enrichment of CD8+ T cells in the tumor core area was positively correlated with the patient’s prognosis, while regulatory T cells (Tregs) tended to accumulate in the tumor periphery area. This pattern is consistent with a close association between tumor-infiltrating lymphocytes and the immunosuppressive microenvironment [83].

ST further revealed the relationship between the spatial location of TILs and their functional status. In patients with a high proportion of intertumoral lymphocytes, natural killer cell-mediated cytotoxicity pathways and T cell receptor signaling pathways are significantly activated in the tumor tissue [84,85]. This phenomenon suggests that ST can provide a new dimension for tumor prognostic assessment by quantifying the spatial distribution of immune cells.

ST can also be used to describe putative interactions between cell types in the immune microenvironment. In TNBC, ST combined with scRNA-seq has shown that the spatial distribution of tumor-associated fibroblasts correlates with patterns of immune-cell infiltration [64,86,87]. For example, FAP+ CAF enrichment at the tumor margin is associated with M2-macrophage polarization and with a T-cell-rejection phenotype, whereas EMILIN1+ CAF accumulation in the tumor core is associated with enhanced CD8+ T-cell infiltration and a more favorable prognosis [88,89]. These associations are correlative rather than causal, and dedicated co-culture and orthotopic studies, such as the work of Chen and colleagues (2025) [41], are an example of the functional follow-up that is needed before targeting these fibroblast subsets clinically.

ST also revealed the spatial specificity of ligand-receptor interactions between immune cells and tumor cells. For example, the high expression of CTLA4 at the interface between tumor cells and Tregs may enable tumors to escape immune surveillance by inhibiting T cell activation [90]. These findings provide a spatial basis for therapeutic strategies that target tumor immune cells.

ST technology also performs exceptionally well in predicting immunotherapy response. The efficacy of immune checkpoint inhibitors (ICIs) is closely related to the spatial characteristics of the immune microenvironment. However, traditional biomarkers, such as the expression of PD-L1, often fail to fully reflect the complexity of the immune microenvironment [91]. By integrating gene expression and spatial location information, ST can accurately identify key immune phenotypes in response to immune checkpoint inhibitors. For example, in HER2-positive breast cancer, ST found that in tumor tissues of patients responding to ICIs, the spatial distribution of tertiary lymphoid structures was associated with the co-localization of CD8+ T cells and dendritic cells [92], while in drug-resistant patients, there was high expression of the immunosuppressive cytokine TGF-β and a lack of immune cell infiltration in the tumor core [34].

ST has also been used to describe spatial features that may underlie immune escape. For example, the aggregation of PD-L1+ macrophages in the tumor core is associated with regions of T-cell exclusion, a pattern that nominates the macrophage-checkpoint axis as a candidate target for the spatially informed deployment of immune-checkpoint inhibitors, pending functional confirmation [90].

In summary (Table 4), spatial transcriptomics provides an unprecedented spatial perspective on the immune microenvironment by elucidating the precise location, interactions, and functional states of immune cells within tumor tissues. This technology has significantly advanced research into the mechanisms of tumor immunotherapy and provided crucial evidence for the development of precision treatment strategies.

### 3.2. Spatial Transcriptomics in Breast Cancer Heterogeneity

Tumor heterogeneity refers to the differences in cell type, gene expression, spatial structure, and microenvironment composition within the same tumor or between different tumors. Breast cancer is a typical heterogeneous tumor, with cancer cells and microenvironments in different regions exhibiting different transcriptional information and biological behaviors [93]. This heterogeneity not only affects tumor growth and metastasis but also determines related treatment responses and prognostic outcomes. Spatial transcriptomics provides a novel tool for studying tumor heterogeneity by integrating gene expression information with spatial information.

A recent high-resolution ST study of luminal, HER2+, and triple-negative breast cancers mapped the spatial heterogeneity of the tumor vasculature. Venous endothelial cells and venous smooth-muscle cells were found to co-localize in spatially restricted domains; this niche is associated with lymphocyte infiltration in a manner consistent with chemokine-mediated recruitment, generating a testable mechanistic hypothesis for enhancing immune infiltration and improving prognosis [54,81]. These observations suggest that, even within a single molecular subtype, differences in spatial architecture, such as vascular topology and matrix–endothelial–immune arrangement, are associated with distinct biological behaviors and therapeutic response potentials [52].

Breast cancer can be classified into several molecular subtypes, including Luminal A, Luminal B, HER2-positive, and triple-negative breast cancer, each with different biological behaviors and clinical outcomes. Spatial transcriptomics technology can reveal the differences in gene expression patterns among different subtypes in tissue space. In 2013, in situ sequencing technology first discovered spatial heterogeneous expression patterns of 31 genes in breast cancer tissue, revealing differences in gene expression between different regions within breast cancer [35]. Subsequently, Svedlund and colleagues used ST technology to perform a molecular–morphological integration analysis of 91 genes in different molecular subtypes of breast cancer, systematically revealing the intratumoral heterogeneity patterns related to subtypes [55]. For example, in triple-negative breast cancer, a subtype of immunomodulatory space has been identified, which is enriched in tertiary lymphoid structures and tumor-infiltrating lymphocytes in the region. This feature is closely associated with significant benefits from immune checkpoint inhibitor therapy [94]. In contrast, the basal-like subtype exhibits molecular characteristics of high proliferation and activation of DNA repair pathways, which are closely related to the tumor’s stronger proliferative capacity and higher malignancy. Table 5 contrasts the conventional bulk-defined molecular subtyping framework with the ST-derived spatial subtyping axis emerging from this body of work.

ST not only describes spatial differences between breast-cancer subtypes but is also associated with clinical prognosis. Integrating ST with histology, one study found that the spatial distribution of tumor plaques is associated with patient outcome, with high-risk patients showing a 2.9-fold elevated risk of death or relapse relative to low-risk patients, highlighting the prognostic value of regional information within a single tumor [35,95]. Coupling ST with deconvolution has further distinguished spatial stromal phenotypes: the stromal compartment of the mesenchymal subtype is associated with an immunosuppressive microenvironment, whereas the stromal compartment of the mesenchymal-stem-cell-like subtype is associated with a pro-angiogenic phenotype, consistent with a regulatory contribution of spatial heterogeneity to treatment response [96]. To resolve cell-type mixtures within ST spots, researchers increasingly integrate ST with scRNA-seq, spatial proteomics, and histology; this multimodal integration improves the assignment of cells to stromal, vascular, and immune compartments and supports inference of their functional status [20,93,97]. Multiple studies have argued that such integration is a necessary step toward the clinical adoption of ST.

### 3.3. Spatial Transcriptomics in Drug Resistance Mechanisms

Deaths and recurrences in breast cancer patients are largely attributed to tumor metastasis and treatment resistance [80]. Conventional bulk RNA-seq can identify averaged resistance-associated programs, and scRNA-seq can resolve resistant cell states after tissue dissociation, but neither approach retains the physical arrangement of malignant clones, stromal cells, immune cells, vessels, and drug-exposed or hypoxic regions within intact tumor tissue. Spatial transcriptomics therefore adds a distinct layer to resistance research: it asks not only which pathway is active, but where the pathway is active, which neighboring cells are present, and whether resistant and sensitive programs coexist within the same lesion [25,35,52].

Several resistance-related pathways have now been examined in the spatial context. In breast cancer ST datasets, drug-response modeling across tumor regions has shown that predicted sensitivity to chemotherapy, endocrine therapy, targeted therapy, and immunotherapy can vary substantially from one spatial domain to another, indicating that therapeutic response is shaped by local tumor-microenvironment organization rather than by a single tumor-wide transcriptional average [98]. Regionally enriched programs include efflux-transporter activity such as ABC-family transporters in poorly infiltrated tumor cores, local xenobiotic or steroid-metabolism programs in peripheral tumor regions, hypoxia and oxidative-phosphorylation programs at invasive or metastatic margins, and immune-checkpoint or TGF-beta-associated immunosuppressive programs in immune-excluded niches [95,98,99,100,101,102]. These pathways are not entirely new to breast cancer biology, but ST refines them by locating them in specific histological neighborhoods and by showing their spatial coexistence with immune exclusion, CAF-rich stroma, vascular niches, or metastatic interfaces.

Compared with non-spatial approaches, the main contribution of ST is the ability to separate tumor-wide abundance from local niche function. Bulk RNA-seq may report high expression of a resistance gene but cannot distinguish whether that signal comes from a small resistant clone, surrounding fibroblasts, macrophages, or endothelial cells. scRNA-seq can identify therapy-associated resistant cell states, such as paclitaxel-resistance programs in TNBC, but loses the in situ contacts that may explain why that state persists [103]. By contrast, ST can place resistant malignant regions next to FAP-positive CAFs, PD-L1-positive macrophages, CCL19/CCL21-producing venular smooth-muscle cells, or tertiary-lymphoid-structure-associated immune aggregates, thereby clarifying whether a resistance program is tumor-cell-intrinsic, microenvironment-driven, or jointly organized by both compartments [25,34,54,70,82].

The strongest current evidence that ST adds genuinely new information comes from spatial drug-response and treatment-response studies. Jimenez-Santos and colleagues integrated 10x Visium profiles with pharmacogenomic prediction and showed tumor microenvironment-driven drug-response heterogeneity and clonal therapeutic heterogeneity within breast tumors [98]. Shiao and colleagues used paired single-cell and spatial profiling in triple-negative breast cancer treated with pembrolizumab plus radiation and identified distinct response trajectories that were linked to spatial immune organization rather than to marker expression alone [82]. In metastatic breast cancer, multi-modal spatial profiling across biopsies from different anatomic sites showed spatial phenotypes of epithelial-to-mesenchymal transition and local T-cell infiltration or exclusion, supporting the idea that resistance and immune escape are organized at the tissue-neighborhood level [25].

**Figure 3:**
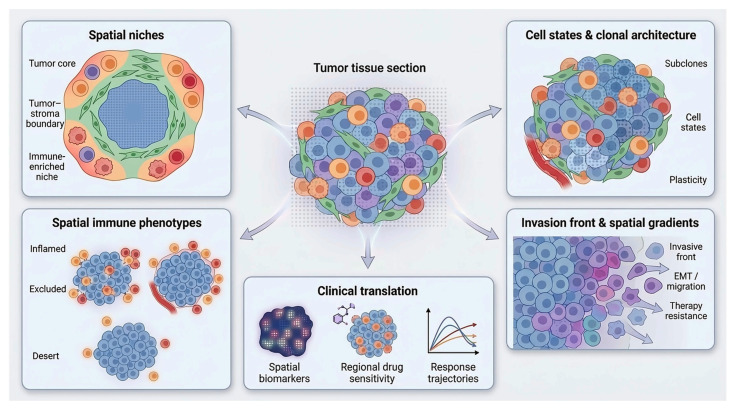
Key biological insights enabled by spatial transcriptomics in breast cancer, including spatial niches, cell states and clonal architecture, spatial immune phenotypes, invasion front and spatial gradients, and clinical translation. Arrows connect the central tumor tissue to each type of insight. Different colors represent different tumor regions, cell populations, immune infiltration patterns, and spatial gradients.

At the same time, these findings should be interpreted as spatially resolved hypotheses rather than definitive resistance drivers. ST can nominate ABC transporter activity, CYP-related metabolism, hypoxia/OXPHOS switching, TGF-beta-mediated immune exclusion, CAF-associated matrix remodeling, and macrophage-checkpoint niches as regionally organized resistance programs, but causal assignment still requires longitudinal biopsies, perturbation studies, orthogonal protein or metabolite validation, and treatment-linked clinical cohorts. Thus, ST has not replaced bulk RNA-seq or scRNA-seq; instead, it complements them by converting resistance signatures into anatomically localized and clinically testable tissue programs. These spatially organized mechanisms are summarized in Figure 3 and Table 6.

## 4. Prospects for Spatial Transcriptomics in Precision Medicine for Breast Cancer

**Figure 4:**
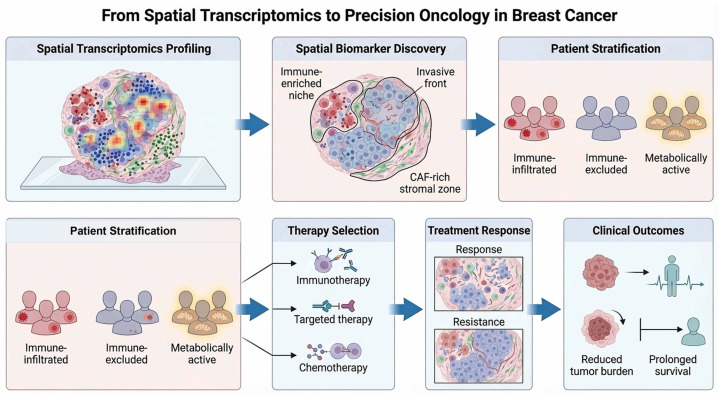
End-to-end workflow from spatial transcriptomics to precision oncology in breast cancer, including tissue profiling, spatial biomarker discovery, patient stratification, therapy selection, treatment response, and clinical outcomes. Arrows indicate the translational workflow from tissue profiling to clinical outcomes. Different colors represent different tumor regions, patient subgroups, and treatment responses.

Spatial transcriptomics provides a new pathway from mechanistic analysis to clinical translation for precision treatment of breast cancer by analyzing tumor spatial heterogeneity and characterizing the interaction networks of microenvironmental cells [104]. Through the deep integration of single-cell sequencing, multi-omics integration, and artificial intelligence technologies, ST is accelerating the transformation of breast cancer diagnosis and treatment from a “population-based” approach to a precise, “individualized” approach [105]. Overall, spatial transcriptomics provides a coherent translational framework that links spatial molecular profiling to breast cancer patient stratification, treatment decisions, and clinical outcomes (Figure 4), and has already demonstrated significant breakthrough potential in the following areas. Figure 4 summarizes the end-to-end pipeline from tissue acquisition to clinical decision-support, providing the visual workflow requested for synthesizing ST data with downstream clinical inference.

### 4.1. Spatial Dimension of Molecular Subtyping

Conventional molecular subtyping of breast cancer assigns tumors to luminal, HER2-positive, or triple-negative classes on the basis of bulk gene-expression averages or IHC surrogates, which limits the visibility of intra-tumoral spatial heterogeneity [56]. ST, by virtue of its near-single-cell spatial resolution, has begun to reveal spatial ecotypes that cut across these conventional classes. For example, ST has described four spatially discrete programs within breast tumors, namely estrogen response, proliferation, hypoxia, and inflammation, with the proliferation program preferentially enriched at the invasion front and positively correlated with the risk of lymph-node metastasis [106]. At the tumor–stromal junction, SREBF1 is specifically and highly expressed, and is associated with lipid-metabolism reprogramming, raising the possibility that it contributes to lymph-node metastasis pending perturbation evidence [46]. These findings motivate the development of spatially guided subtyping markers and offer a new paradigm for selecting patients likely to benefit from chemotherapy or targeted therapy [102]. A large clinical-cohort study applied ST to pathological sections from more than 1000 breast-cancer patients, inferred the composition of the TME and the spatial gene-expression characteristics of each tumor, and nominated three new spatial-driven subtypes with significantly different survival profiles [107].

### 4.2. Spatial Dissection of Drug Resistance Mechanisms

For precision medicine, ST is most useful when it is treated as a patient-stratification and hypothesis-generation layer rather than as stand-alone proof of drug resistance. The clearest example is spatial drug-response modeling, in which Visium profiles from breast tumors were linked to predicted sensitivity for more than 1200 agents; this analysis showed that the same tumor can contain neighboring domains with different predicted responses to chemotherapy and targeted agents [98]. Spatial and single-cell analyses of mitochondrial calcium uniporter expression also suggest that metabolic and immune features can be combined to nominate breast cancer subsets for follow-up therapeutic testing [101,108,109].

For HER2-positive and triple-negative disease, ST can further identify where resistant or immune-excluded states occur relative to CAFs, macrophages, venular niches, and TLS-like immune aggregates [25,34,54,70,82]. Non-spatial therapeutic studies, including CD47/HER2 bispecific-antibody work in trastuzumab- or radioresistant HER2-positive breast cancer, provide mechanistic candidates that ST could help localize in tissue context [110,111]. These observations are clinically relevant because they can help prioritize where to sample tissue, which combinations deserve functional validation, and which patients may warrant spatially informed companion assays. However, the manuscript now avoids presenting ST-derived spatial associations as already validated resistance drivers; each candidate pathway still requires perturbation and prospective clinical confirmation.

### 4.3. ST in Cancer Diagnosis and Treatment

It is worth noting that the deep integration of spatial transcriptomics with multimodal technologies such as spatial proteomics, metabolomics, and radiomics is gradually becoming an important direction for future cancer research [57]. For example, the 10x Genomics Visium platform, in conjunction with scRNA-seq, has been used to analyze the metabolic evolution of the pre-metastatic microenvironment in breast cancer and to show that oxidative-phosphorylation genes are enriched at the edge of metastatic lesions, motivating the development of targeting strategies [73,112]. In preclinical settings, ST-based maps of CAF distribution are guiding viral and cellular therapies directed against FAP-high CAFs, combined with PD-1/PD-L1 inhibitors, to enhance local immune activation and tumor regression in TNBC models [41,68]. A recent study showed that combining ST with histological images allows a convolutional neural network (CNN) to predict local gene expression from haematoxylin and eosin slides: trained on 23 breast-cancer patients and 30,612 spatial spots, the model predicted the expression of multiple breast-cancer biomarkers at 100 µm resolution and generalized to external cohorts such as TCGA [104]. Such work suggests that spatial gene expression can be inferred from routine pathology slides using deep-learning-based computational pathology, effectively expanding cohort size and accelerating translation of spatial omics into clinical workflows [105]. As ST continues to improve in FFPE compatibility, workflow standardization, and cost, it is expected to be integrated progressively into routine pathology [58], and ultimately to contribute to reshaping the diagnostic and therapeutic paradigm for breast cancer.

Two concrete clinical-decision pathways illustrate where this integration is closest to deployment. First, ST-supported quantification of tertiary lymphoid structures (TLS) can serve as a companion to PD-L1 IHC for immune-checkpoint-inhibitor stratification: in patients with intermediate or discordant PD-L1 readouts, the presence, density, and CD8+ T-cell-dendritic-cell composition of TLS quantified by ST may refine the predicted likelihood of benefit from ICI therapy, particularly in HER2+ and triple-negative disease. Second, ST-supported spatial scoring of CAF subtypes, in particular the abundance and tumor-margin localization of FAP+ CAFs relative to EMILIN1+ CAFs, may stratify HER2+ patients at risk of trastuzumab resistance and identify those in whom CAF-directed combination regimens, such as FAP-targeted cell therapy plus checkpoint blockade, warrant prospective evaluation. Both pathways position ST as a companion-diagnostic or decision-support layer rather than a stand-alone primary diagnostic.

Consistent with the field’s broader assessment that routine pathology implementation remains multi-year on the horizon, no ST assay has, at the time of writing, received regulatory clearance for routine breast-cancer diagnostics. The realistic translation timeline depends on three convergent advances: harmonized standardization of platforms, probes, and analytic pipelines; maturation of FFPE-compatible workflows so that archival pathology blocks can be used at scale; and prospective clinical validation in adequately powered cohorts with predefined decision rules. Within this timeline, companion-diagnostic and decision-support roles, in which ST refines stratification within an established histopathology-driven workflow, are reachable earlier than a primary diagnostic role in which ST replaces existing assays.

## 5. Current Limitations of Spatial Transcriptomics

Despite the substantial advances surveyed above, spatial transcriptomics (ST) remains a young technology whose findings, validation pathways, computational pipelines, and clinical-grade workflows are still maturing. In this section we provide a deliberately critical synthesis of the principal limitations that, in our view, must be acknowledged and addressed before ST can be incorporated into routine breast-cancer diagnostics and decision-making. We organize the discussion under four headings: (i) the epistemological gap between spatial correlation and functional causality; (ii) computational and benchmarking challenges; (iii) clinical translation and regulatory considerations; and (iv) concrete routes for innovation that we believe the field should prioritize.

### 5.1. From Spatial Correlation to Functional Causality

ST measurements are, by construction, associative. A spatial transcriptome captures the steady-state abundance and location of transcripts in a fixed tissue section, but does not, on its own, establish that a given gene, cell state, or niche is causally responsible for the phenotype with which it is spatially co-localized. Throughout the breast-cancer literature reviewed above, several spatial associations have been reported with strong causal-sounding language; in the present review, we deliberately softened such verbs to associative formulations, and we recommend that readers interpret any ST-derived claim as a hypothesis that requires orthogonal validation. Three lines of follow-up are particularly important: targeted perturbation experiments that establish loss- or gain-of-function in the implicated cells, longitudinal sampling that distinguishes causes from consequences, and orthogonal-omics confirmation that anchors the transcriptional signal to functional protein or metabolite output.

A practical experimental toolbox is now available for converting ST hypotheses into causal claims. Perturb-FISH and image-based Perturb-MERFISH couple pooled CRISPR perturbations with high-multiplex single-cell spatial readout, enabling thousands of genetic perturbations to be queried in their native spatial context [113]. Lineage-traced ST in genetically engineered mouse models of breast cancer permits clones to be followed across space and time, distinguishing tumor-cell-intrinsic from microenvironment-driven phenotypes. Patient-derived organoids and explants can be co-cultured with stromal or immune components, profiled by ST, and then subjected to targeted perturbation to interrogate niche dependencies in a human-derived setting [114]. Finally, ST-guided in vivo CRISPR screens, in which candidate drivers identified by ST are prioritized for pooled functional screening in orthotopic or syngeneic mouse models, can close the loop from observation to causal mechanism [115].

Advanced metabolic and chemical imaging modalities further complement ST by reporting on functional outputs that mRNA abundance cannot. Mass spectrometry imaging (MSI), including matrix-assisted laser-desorption–ionization MSI (MALDI-MSI) and desorption electrospray ionization MSI, maps lipid, metabolite, and drug distributions on the same tissue sections that can be profiled by ST [45]. Stimulated Raman scattering (SRS) microscopy provides label-free, chemically specific imaging of lipid and protein content at sub-micron resolution, and can be co-registered with ST data. Isotope-tracing imaging, in which 13C- or 15N-labeled substrates are infused into patient explants or mouse models prior to ST and MSI, allows pathway flux to be inferred in the spatial context [116].

We note, finally, that several molecular-biophysical techniques sometimes mentioned alongside ST—including surface plasmon resonance, isothermal titration calorimetry, and single-particle cryo-electron microscopy—operate at the molecular and biomolecular scale and are not, properly speaking, tissue-scale spatial methods. They remain essential for mechanistic characterization of individual protein–ligand or protein–protein interactions identified by ST, but should not be regarded as substitutes for the orthogonal in situ approaches discussed above when the question is whether a spatial correlation reflects a causal tissue-scale mechanism.

### 5.2. Computational and Benchmarking Challenges

The analytical stack on which ST findings depend introduces its own set of limitations that are often under-reported. Cell-type deconvolution—the inference of cell-type proportions from sequencing-based ST spots that contain multiple cells—is performed by a family of algorithms, including Robust Cell Type Decomposition (RCTD), Conditional Autoregressive-based Deconvolution (CARD), Cell2location, SpotLight, and Tangram [117,118,119]. The accuracy of these methods is strongly dependent on the choice and completeness of the single-cell reference, degrades at low spot capture (a particular concern for capture-based platforms applied to FFPE tissue), and cannot, at present, be evaluated against an accepted ground truth for breast-cancer tissue.

Spatial-domain detection—the partitioning of tissue into transcriptionally coherent regions—is similarly dependent on algorithmic choices. BayesSpace, STAGATE, SpaGCN, GraphST, and SEDR each impose different assumptions on the spatial graph used to share information between neighboring spots, and the resulting domain calls can be sensitive to graph-construction parameters such as the number of nearest neighbors, the smoothing strength, and the underlying spot resolution [120,121]. Validation of spatial domains against histopathology is rarely performed at the level of individual breast-cancer subtypes, and the difficulty of reconciling algorithmic domain boundaries with pathologist-annotated regions remains a barrier to clinical interpretation.

The integration of scRNA-seq with ST is essential for both deconvolution and label transfer, and is supported by tools such as Harmony, scVI/scANVI, STAligner, and PASTE. These methods correct for batch effects between modalities and align datasets in a shared latent space, but they propagate two characteristic failure modes: batch effects between scRNA-seq and ST that are confounded with biological signal, and label-transfer errors that are inflated when the reference single-cell atlas is incomplete for rare immune or stromal subsets relevant to breast cancer. Both failure modes feed forward into downstream deconvolution and spatial-domain calls, and we recommend that downstream conclusions be tested for robustness across multiple reference atlases and integration strategies before they are advanced as biological claims.

Benchmarking datasets remain the most basic missing infrastructure. The systematic comparison of sequencing-based ST platforms reported by You and colleagues [26] provides an important reference point, but, as the authors themselves note, this benchmark covers platform-level performance and does not extend to the full analytical stack of deconvolution, spatial-domain detection, and modality integration. No accepted end-to-end benchmark exists for breast-cancer ST that spans platform choice, deconvolution algorithm, integration strategy, and clinical outcome. We recommend that a consortium-level breast-cancer benchmark—analogous in spirit to the MAQC effort for bulk RNA sequencing—be developed, with shared reference tissues, paired scRNA-seq and ST data, and harmonized pathology annotations, so that algorithmic choices in the field can be evaluated against a common standard.

### 5.3. Clinical Translation and Regulatory Considerations

Clinical-grade deployment of ST will require closure of several pre-analytical, analytical, economic, and regulatory gaps that are not, at present, well-characterized in the literature [122]. Pre-analytical variability is the most basic concern: capture efficiency and transcript integrity in ST are sensitive to whether tissue is fresh-frozen or formalin-fixed paraffin-embedded, to ischemia time between excision and fixation, to fixation chemistry and duration, to section thickness, and—for capture-based protocols—to RNA-integrity-number thresholds that vary between platforms and protocols [15,26,105]. The breast-cancer community has begun to harmonize pre-analytical handling for bulk and scRNA-seq, but no consensus pre-analytical standard yet exists for ST.

Inter-laboratory standardization gaps amplify this variability. There is, at present, no consensus quality-control metric for an ST experiment that is reported uniformly across the principal platforms; no reference tissue block circulated between laboratories for cross-platform benchmarking; and no minimum-information-for-ST reporting standard analogous to MIQE for quantitative PCR or MIAME for microarrays [16,26]. As a result, comparison of results across laboratories and across platforms is generally performed on an ad hoc basis, which is inadequate for clinical adoption.

Cost-per-section and throughput are further considerations that bear directly on clinical scalability. Sequencing-based ST and high-multiplex imaging-based ST currently cost substantially more per section than bulk RNA sequencing or single-cell RNA sequencing of dissociated material, although peer-reviewed cost values are scarce and vary substantially with platform, panel size, and sequencing depth [14,15,26]. We do not give explicit per-section price estimates here because we are not aware of peer-reviewed sources that would allow us to do so reliably; we instead note qualitatively that the current cost differential is approximately one order of magnitude relative to bulk RNA-seq for capture-based ST, and somewhat larger for high-multiplex imaging-based ST.

At the time of writing, no spatial transcriptomics assay has received CE-IVD marking or U.S. FDA clearance for routine clinical use, and no clinical-grade companion-diagnostic ST product is, to our knowledge, in late-stage regulatory submission [57,105]. Any clinical-grade ST assay will need to satisfy the standard analytical-validity, clinical-validity, and clinical-utility framework: analytical validity (accuracy, precision, reproducibility of the measurement), clinical validity (the demonstrated association between the measurement and a clinical phenotype), and clinical utility (the demonstrated impact of the measurement on patient outcomes). Each of these layers requires multi-center prospective validation studies with pre-registered analytical pipelines and central pathology review, and the regulatory timeline for the first cleared spatial assays is therefore best measured in years rather than months.

We propose three concrete standardization paths that the field could adopt to accelerate this timeline. First, an international ST quality-control consortium—modeled on the MAQC initiative for bulk RNA sequencing—should be established to develop reference tissues, standard analytical pipelines, and reporting templates [26]. Second, FFPE-compatible Visium-style assays, which already integrate naturally with the existing clinical pathology workflow, should be prioritized as the regulatory bridge between research-grade and clinical-grade ST [15,105]. Third, hybrid evidence packages that combine retrospective archival cohorts with prospective validation arms should be designed in collaboration with regulators from the outset, so that approval requirements are clear before validation studies are launched.

### 5.4. Routes for Innovation

Three routes for innovation appear, in our view, particularly well-aligned with the field’s current limitations and with the clinical-translation gaps described above. First, a cost reduction is achievable through the patterned-array printing of capture probes at higher density and lower cost, and through reduced sequencing-depth protocols that exploit modern deconvolution algorithms to recover cell-type signals from sparser reads [26,117,118,119]. Both directions are technically tractable and would substantially improve the cost-per-section calculus described in Section 5.3.

Second, AI-predicted ST from hematoxylin-and-eosin (H&E)-stained whole-slide images offers a regulatory-friendly companion modality. Convolutional and transformer-based models trained on paired H&E + ST datasets can predict spatial gene expression directly from routine pathology slides, enabling spatial inference at the scale of existing clinical archives without additional wet-lab cost [56,104,107,123]. We return to this direction in Section 6, where we discuss it as a near-term opportunity for AI-driven precision medicine.

Third, federated multi-site ST cohorts—in which raw data remain at each site and only model updates are shared—offer a route to assembling breast-cancer cohorts of clinically meaningful size while preserving patient privacy and reducing population and batch biases. Such federated designs are particularly well-suited to the international, multi-center validation studies that will be required for regulatory clearance, and we encourage their early adoption in breast-cancer ST consortia [105].

## 6. Future Perspectives

Looking ahead, we identify three coordinated directions that, if pursued together, are likely to define the next phase of spatial-transcriptomics research in breast cancer: emerging spatial modalities, AI strategies, and longitudinal study designs. We then close the section with a brief discussion of the training, infrastructure, and collaboration models that will be required to support these directions in practice.

Emerging Spatial Modalities: The first direction concerns the technology platforms themselves. Live-cell spatial transcriptomics—including live-FISH, live MERFISH variants, and emerging label-free Raman-based readouts—promises to extend ST from a fixed-tissue snapshot to a time-resolved measurement of transcriptional dynamics in living tissue, at the price of a substantial reduction in gene-panel size [124]. Spatial epigenomics—spatial-ATAC-seq, MISAR-seq (joint spatial RNA + ATAC), spatial-CUT&Tag for histone modifications, and emerging spatial DNA-methylation assays—is likely to be particularly informative for breast-cancer subtype plasticity and treatment-induced cell-state transitions, where regulatory rather than transcriptional changes often drive phenotype [49,50,51]. Subcellular-resolution ST platforms—Visium HD, Xenium 5K, and Stereo-seq—are now approaching single-cell and even subcellular resolution at transcriptome scale, eroding the historical resolution-versus-coverage trade-off and enabling spatial analysis of intracellular RNA compartmentalization in breast-cancer tissue.

AI Strategies: The second direction concerns analytical methods. AI-based inference of spatial gene expression from routine H&E-stained whole-slide images—exemplified by the recent work of Shulman and colleagues [107]—has the potential to bring spatial-omics information to bear on archival cohorts that have already accumulated decades of clinical follow-up, without the cost and pre-analytical constraints of running ST itself. Spatial foundation models trained on large multi-platform ST corpora are emerging as general-purpose representations for downstream tasks, as recently reviewed by Liu and colleagues [57]. Causal-inference deep learning—methods that combine longitudinal or perturbational data with structured causal priors—may help bridge the correlation-to-causation gap discussed in Section 5.1, and AI-driven prioritization of therapeutic targets from spatially resolved tumor–immune interactions is an active area in which we anticipate substantial progress in the next two to three years [107]. More broadly, recent work on AI-enabled drug-target discovery and AI-supported nanomedicine or organ-specific therapy provides a complementary computational context for translating spatially resolved biomarkers into prioritized therapeutic hypotheses [125,126].

Longitudinal Study Designs: The third direction concerns clinical study design. Most published breast-cancer ST studies are cross-sectional, profiling a single resection per patient; the field’s most important future experiments are paired pre-treatment and on-treatment biopsy ST cohorts, in which the same patient is sampled at clinically meaningful time points and the spatial response to therapy is quantified at the niche level. Serial liquid-biopsy profiling integrated with serial ST of tumor tissue offers a route to monitor the spatial trajectory of treatment response and resistance with reduced patient burden [127]. Early-phase clinical trials should incorporate spatial-transcriptomics endpoints as pre-specified secondary or exploratory measures, so that the clinical-utility evidence base required for regulatory clearance (Section 5.3) accumulates in parallel with the trials themselves. The Shiao and colleagues’ [82] study of pembrolizumab plus radiation in triple-negative breast cancer, in which response trajectories were defined from paired single-cell and spatial profiles, is an example of the longitudinal design we have in mind.

Realizing these directions will require investment beyond the technology itself. The most immediate need is for cross-trained pathologist-bioinformatician teams who can interpret spatial data in their clinical context and design analytical pipelines that respect the pathology workflow. Infrastructure requirements include high-throughput slide scanners, validated cryo-storage and FFPE archives, and high-memory compute environments capable of processing the multi-terabyte datasets that subcellular-resolution ST generates. Interdisciplinary collaboration—between pathology, oncology, computational biology, biomedical engineering, and regulatory science—will be essential, and we encourage breast-cancer research consortia to formalize such collaborations as part of grant and trial design rather than leave them to ad hoc arrangements.

## 7. Conclusions

This review sought to provide a comprehensive but critical synthesis of spatial transcriptomics in breast cancer. We surveyed four substantive contributions of the field. First, we outlined the technology landscape—imaging-based, capture-based, in situ sequencing, and spatial multi-omics platforms—and placed ST in the context of complementary spatial modalities including spatial proteomics and metabolomics. Second, we synthesized the breast-cancer biological insights that ST has enabled, particularly the spatial dissection of tumor-microenvironment ecology, intratumoral heterogeneity, and tumor–immune interactions. Third, we reviewed the application of ST to the dissection of drug-resistance mechanisms across the major treatment classes used in breast cancer. Fourth, we outlined the precision-medicine outlook in which the ST findings are translated into spatially guided molecular subtyping, targeted-therapy selection, and decision-support tools.

At the same time, our review identified three principal critical issues that the breast-cancer ST community must address. The first is the absence of standardization: there is, at present, no consensus pre-analytical protocol, no shared quality-control metric, and no minimum-information reporting standard for ST experiments, which makes results across laboratories and platforms difficult to compare. The second is the gap between spatial correlation and biological causality: most published ST findings are associative, and the field has yet to systematically incorporate the perturbational, longitudinal, and orthogonal-omics follow-up that would convert spatial observations into causal claims. The third is the regulatory pathway: no spatial assay has yet been cleared for routine clinical use, and the analytical-validity, clinical-validity, and clinical-utility evidence that regulators require for breast-cancer companion diagnostics has not yet been assembled.

Taken together, these contributions and limitations suggest that spatial transcriptomics is on a credible path toward companion-diagnostic and decision-support roles in precision oncology; routine primary-diagnostic use will require the field to honestly acknowledge and address the methodological and translational limitations summarized above. In our view, the most productive way for the breast-cancer community to accelerate this trajectory is to invest jointly in standardization infrastructure, in causal-inference experimental designs, and in early engagement with regulators, so that the analytical and clinical evidence required for clearance accumulates alongside, rather than after, the next generation of biological discoveries.

## Figures and Tables

**Table 1:** Comparative summary of major spatial transcriptomics platforms applied to breast cancer research. Resolution and gene-capture values reflect manufacturer specifications and peer-reviewed benchmarking studies available at the time of writing.

No.	Platform	Resolution	Genes Captured	FFPE Compatibility	Strengths	Limitations	Representative Breast-Cancer Study
1	10x Visium	55 μm spot diameter, 100 μm center-to-center (~1–10 cells per spot)	Whole transcriptome (~18,000–20,000 genes)	Yes (FFPE v2 probe-based assay)	Commercial, mature pipeline; FFPE-archive compatible	Multi-cell spot resolution; requires deconvolution	Wu SZ, Nat Genet 2021;53:1334 [52]
2	10x Visium HD	2 μm bin (binnable to 8 μm/16 μm)	Whole transcriptome	Yes (FFPE supported)	Near-single-cell resolution at whole-transcriptome scale	Released 2024; high sequencing cost	Oliveira MF, Nat Genet 2025;57:1512 [8]
3	Slide-seq v2	10 μm bead	Whole transcriptome (lower sensitivity than Visium per bead)	No (fresh-frozen only)	Near-cellular resolution; open-source bead chemistry	Lower capture efficiency per bead; limited commercial support	Stickels RR, Nat Biotechnol 2021;39:313 [32]
4	DBiT-seq	10–25 μm (microfluidic channel width)	Whole transcriptome + optional protein co-detection	Compatible with formaldehyde-fixed sections	Native multi-omics via deterministic barcoding	Custom microfluidic device required; throughput limited	Liu Y, Cell 2020;183:1665 [30]
5	MERFISH	~100 nm (sub-cellular)	Targeted panel of 100–1000 genes	Limited (fresh-frozen primary; FFPE protocols emerging)	Sub-cellular resolution; single-molecule sensitivity	Pre-designed panel; long imaging times	Moffitt JR & Zhuang X. Methods Enzymol 2016;572:1 [18]
6	seqFISH+	~200 nm (sub-cellular)	Up to ~10,000 genes via sequential barcoding	Fresh-frozen primary	High plex with single-molecule sensitivity	Specialized microscopy; throughput-limited	Eng CL, Nature 2019;568:235 [53]
7	10x Xenium	~200 nm (sub-cellular, single-cell segmentation)	Pre-designed panels (280-plex or 5000-plex)	Yes (FFPE supported)	Automated FFPE workflow; validated breast panel	Targeted, not whole-transcriptome; instrument cost	Wang X, Nat Commun 2025;16:3348 [54]
8	Stereo-seq	~500 nm spot (binnable)	Whole transcriptome	Fresh-frozen primary; FFPE protocols emerging	Centimeter-scale field of view at sub-cellular resolution	Limited commercial availability; high sequencing depth	Chen A, Cell 2022;185:1777 [29]
9	ISS/HybISS	~200–500 nm (sub-cellular)	Targeted panels (tens to hundreds of genes)	FFPE-compatible	Direct in situ sequencing on pathology sections	Limited plex; long imaging cycles	Svedlund J, EBioMedicine 2019;48:212 [55]
10	Spatial-CUT and Tag/spatial-ATAC	~20–50 μm (DBiT-style microfluidic)	Genome-wide histone modifications or chromatin accessibility	Fresh-frozen	Adds spatial epigenomics; regulatory-state mapping	Early-stage technology; limited BC applications	Deng Y, Science 2022;375:681 [50]
11	AI-predicted ST (H&E → ST)	Tile-level (~50–100 μm), inferred from histology	Whole-transcriptome inference	Yes (operates on standard FFPE H&E slides)	No additional wet-lab cost; scalable to existing archives	Predictions are inferential; require careful validation	He B, Nat Biomed Eng 2020;4:827 [56]

Abbreviations: BC, breast cancer; FFPE, formalin-fixed paraffin-embedded; HD, high-definition; ISS, in situ sequencing; HybISS, hybridization-based in situ sequencing; ST, spatial transcriptomics.

**Table 2:** Complementary spatial transcriptomic and spatial proteomic modalities used in breast cancer. ST profiles RNA at variable resolution and gene depth (see Table 1 for platform-level detail), whereas spatial proteomics (IMC, CODEX/PhenoCycler, MIBI) profiles 40–60 protein analytes at sub-cellular resolution on FFPE sections.

Modality	Target	Resolution	Representative Breast-Cancer Study
Spatial transcriptomics (capture- or imaging-based)	mRNA (whole-transcriptome or targeted panel)	0.2–100 μm depending on platform (see Table 1)	Wu SZ, Nat Genet 2021;53:1334 [52]
Imaging mass cytometry (IMC/Hyperion)	Protein (~40-plex metal-tagged antibodies)	~1 μm	Jackson HW, Nature 2020;578:615 [59]
CODEX/PhenoCycler	Protein (~40–60-plex DNA-barcoded antibodies, FFPE-compatible)	Sub-cellular (~0.3–0.5 μm)	Schürch CM, Cell 2020;182:1341 [60]
MIBI/MIBI-TOF	Protein (~40-plex metal-tagged antibodies, FFPE-compatible)	~400 nm	Risom T, Cell 2022;185:299 [33]

Abbreviations: IMC, imaging mass cytometry; CODEX, co-detection by indexing; MIBI, multiplexed ion beam imaging; ST, spatial transcriptomics; FFPE, formalin-fixed paraffin-embedded.

**Table 3:** Spatial niches in the breast-cancer tumor microenvironment described by ST to date. These niches are operational descriptions, not formally defined disease entities; their causal contributions to clinical behavior remain to be established by perturbation experiments and prospective clinical studies.

Niche	Location	Dominant Cell Populations	Key Molecular Signals	Putative Role
Invasion-front niche	Tumor–stroma interface	FAP+ iCAF-like fibroblasts; EMT-high tumor cells	FAP, EMT-program genes	Locally associated with invasion and EMT
Immune-exclusion niche	Tumor core	M2-polarized macrophages; cytotoxic-T-cell-depleted regions	PD-L1, CTLA4	Associated with local immune evasion
Perivascular niche	Around small vessels	Venous endothelial cells; venous smooth-muscle cells	Vascular and chemokine cues	Accompanies lymphocyte infiltration into the tumor
Metabolic margin niche	Tumor margin	OXPHOS- and lipid-metabolism-skewed cancer cells	LDHA, lipid-metabolism genes	Spatially associated with metastatic dissemination

Abbreviations: iCAF, inflammatory cancer-associated fibroblast; FAP, fibroblast activation protein; EMT, epithelial–mesenchymal transition; OXPHOS, oxidative phosphorylation; PD-L1, programmed death-ligand 1; CTLA4, cytotoxic T-lymphocyte-associated protein 4; LDHA, lactate dehydrogenase A.

**Table 4:** Functional Role and Case Studies of Spatial Transcriptomics in Breast Cancer Research.

Advantages	Specific Manifestations
Decoding Spatial Heterogeneity	SREBF1 is highly expressed in the tumor–stromal junction of IMPC and is associated with lipid metabolism reprogramming and lymph node metastasis; functional validation is required.
Multi-cell interaction visualization	iCAF subsets spatially co-localize with immunosuppressive cytokines; this co-localization nominates candidate mechanisms of tumor invasion that require functional validation.
Functional gene mapping	CTLA4/PD-L1 accumulates in the tumor core and is spatially associated with T-cell rejection.
Technological integration and innovation	GEO-seq combined with ST assays identifies transitional cell populations at the tumor–immune border.
Immunophenotyping	Quasi-identification of responses to ICIs revealed the spatial distribution patterns of tumor-infiltrating lymphocytes.

Abbreviations: ST, spatial transcriptomics; IMPC, invasive micropapillary carcinoma; iCAF, inflammatory cancer-associated fibroblast; SREBF1, sterol regulatory element-binding transcription factor 1; CTLA4, cytotoxic T-lymphocyte-associated protein 4; PD-L1, programmed death-ligand 1; ICI, immune checkpoint inhibitor; GEO-seq, geographic position sequencing.

**Table 5:** Conventional molecular subtyping versus ST-derived spatial subtyping in breast cancer. ST-derived spatial subtypes provide a complementary axis to bulk-defined molecular subtypes rather than replacing them.

Dimension	Conventional Molecular Subtyping (Bulk + IHC)	ST-Derived Spatial Subtyping
Data source	Bulk transcriptomic/IHC averages over the whole tumor	Spatially resolved transcriptomes within a tissue section
Unit of analysis	Whole tumor	Intra-tumoral ecotypes/niches
Underlying assumption	Intra-tumoral homogeneity	Recurrent spatial co-occurrence of cell states across patients
Current categories	Luminal A, Luminal B, HER2+, triple-negative	Estrogen-response, proliferative, immune-rich, mesenchymal, hypoxic ecotypes
Key spatial observation	Subtype assigned as a single tumor-level label	Multiple programs can co-occur within the same tumor
Clinical maturity	Established prognostic and predictive use	Prognostic and predictive value emerging; standardization pending
Relationship to the other axis	Mature, widely adopted	Complementary axis to molecular subtyping; does not replace it

Abbreviations: IHC, immunohistochemistry; HER2, human epidermal growth factor receptor 2; ST, spatial transcriptomics.

**Table 6:** Region-specific resistance programs within a single breast tumor described by ST. Specific drug classes are illustrative examples; the same molecular program may affect multiple drug classes depending on tumor subtype.

Tumor Region	Resistance Program	Key Molecule/Pathway	Predominantly Affected Drug Class
Tumor core	Drug efflux	ABCB1 transporter	Chemotherapy
Periphery	Local drug metabolism	CYP3A4 enzymatic activity	Endocrine therapy
Invasion front	Metabolic switching	OXPHOS and lipid-metabolism reprogramming	Targeted agents

Abbreviations: ABCB1, ATP-binding cassette sub-family B member 1; CYP3A4, cytochrome P450 3A4; OXPHOS, oxidative phosphorylation.

## Data Availability

No new data were created or analyzed in this study. Data sharing is not applicable to this article.

## Abbreviations

The following abbreviations are used in this manuscript:

ABCB1	ATP-binding cassette sub-family B member 1
AI	artificial intelligence
BC	breast cancer
CAF	cancer-associated fibroblast
CARD	conditional autoregressive-based deconvolution
CE-IVD	Conformité Européenne—In Vitro Diagnostic marking
CNN	convolutional neural network
CODEX	co-detection by indexing (also marketed as PhenoCycler)
CRC	colorectal cancer
CTLA4	cytotoxic T-lymphocyte-associated protein 4
CYP3A4	cytochrome P450 3A4
EMT	epithelial–mesenchymal transition
ER	estrogen receptor
FAP	fibroblast activation protein
FDA	U.S. Food and Drug Administration
FFPE	formalin-fixed paraffin-embedded
GEO-seq	geographic position sequencing
H&E	hematoxylin and eosin
HD	high-definition
HER2	human epidermal growth factor receptor 2
HybISS	hybridization-based in situ sequencing
iCAF	inflammatory cancer-associated fibroblast
ICI	immune checkpoint inhibitor
IHC	immunohistochemistry
IMC	imaging mass cytometry
IMPC	invasive micropapillary carcinoma
ISH	in situ hybridization
ISS	in situ sequencing
IVDR	In Vitro Diagnostic Regulation (EU 2017/746)
LDHA	lactate dehydrogenase A
MIBI	multiplexed ion beam imaging
MISAR-seq	microfluidic indexing-based spatial ATAC and RNA sequencing
OXPHOS	oxidative phosphorylation
PD-L1	programmed death-ligand 1
RCP	rolling-circle product
RCTD	robust cell type decomposition
scRNA-seq	single-cell RNA sequencing
smFISH	single-molecule fluorescence in situ hybridization
SREBF1	sterol regulatory element-binding transcription factor 1
ST	spatial transcriptomics
TME	tumor microenvironment
TNBC	triple-negative breast cancer
